# Efficient Method for High-Throughput Screening of Compound Libraries Targeting Human PD-L1

**DOI:** 10.1021/acsomega.5c12417

**Published:** 2026-03-30

**Authors:** Mohammad Reza Zamani, Kateřina Čermáková, Martin Hadzima, Pavel Šácha, Jan Konvalinka

**Affiliations:** † Charles University, Faculty of Science, Department of Cell Biology, Viničná 7, 12800 Prague 2, Czech Republic; ‡ 89220 Institute of Organic Chemistry and Biochemistry, Czech Academy of Sciences, Flemingovo n. 2, 16610 Prague 6, Czech Republic

## Abstract

The PD-1/PD-L1 immune checkpoint is a pivotal target for cancer immunotherapy. Monoclonal antibodies (mAbs) targeting the PD-1/PD-L1 interaction have achieved clinical success but face limitations, including high production costs, suboptimal tumor penetration, and potential immunogenicity. To address these challenges, we present the DNA-linked Inhibitor Antibody Assay (DIANA)—a robust, high-throughput screening platform optimized for identifying and characterizing low-molecular-weight inhibitors of human PD-L1. DIANA integrates competitive binding with qPCR detection, enabling single-well determination of dissociation constants (*K*_d_) and rapid screening of thousands of compounds. The assay was validated using three FDA-approved mAbs (atezolizumab, avelumab, and durvalumab), the PD-L1-binding macrocyclic peptide WL12, and the native PD-1 receptor, yielding *K*_d_ values consistent with the literature. DIANA demonstrated a broad dynamic range spanning more than 4 orders of magnitude, excellent robustness (*Z*′-factor = 0.94), and high tolerance to DMSO (up to 10%). We applied DIANA to screen two libraries: a 5,280-compound in-house library (pooled format) and a 1,298-compound commercial peptidomimetic library (individual format). While very weak initial hits were detected, none were confirmed in follow-up manual (non-HTS) experiments or in an orthogonal cell-based assay. Nonetheless, DIANA’s sensitivity, scalability, and minimal sample requirements establish it as a powerful tool for accelerating the discovery of next-generation PD-1/PD-L1 inhibitors and overcoming key limitations of conventional screening methods.

## Graphical abstract

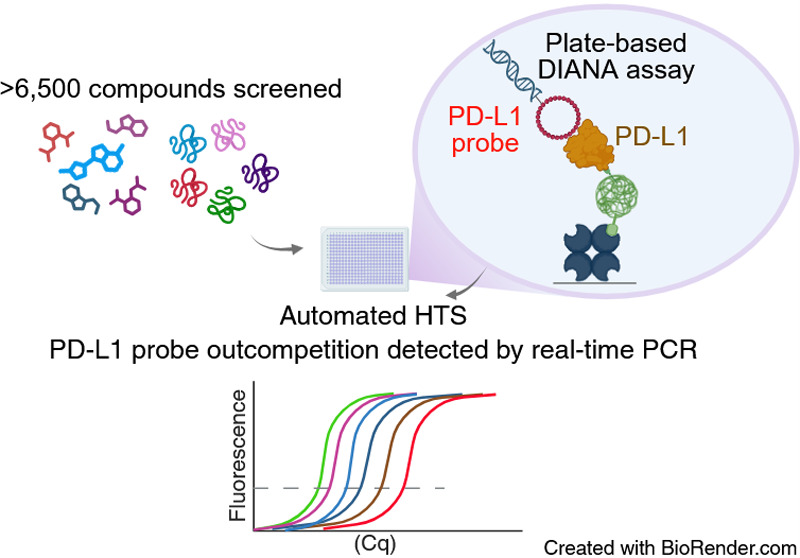

## Introduction

PD-L1 is a transmembrane protein expressed by immune cells, including T and B cells, macrophages, and dendritic cells.^1^ It plays a crucial role in maintaining immune tolerance by interacting with PD-1, preventing excessive immune activation that could lead to inflammation and autoimmunity.^2^ However, tumor cells can exploit this pathway by expressing PD-L1, allowing them to evade immune detection and suppress antitumor responses.^3^

To counteract this immune evasion, researchers have developed monoclonal antibodies (mAbs) that target the PD-1/PD-L1 interaction and reactivate the immune system to attack cancer cells. Several mAbs, including atezolizumab, durvalumab, and avelumab, have been approved by the U.S. FDA for the treatment of several cancers.^4^ While effective, these therapies have limitations such as complex and costly production, suboptimal tumor penetration, and potential immunogenicity.^5^

Alternative approaches, including low-molecular-weight (LMW) compounds, are being explored to overcome these challenges, but none have yet matched the efficacy of mAbs. As a result, the search for more effective PD-1/PD-L1 inhibitors through advanced high-throughput screening (HTS) methods remains a critical area of research in cancer immunotherapy.

Surface plasmon resonance (SPR) has been used to study binding interactions, but its application in high-throughput screening (HTS) is limited by throughput and the need for protein immobilization.^6^ Other techniques, including isothermal titration calorimetry (ITC),^7^ biolayer interferometry (BLI),^8^ differential scanning fluorometry (DSF),^9^ microscale thermophoresis (MST),^10^ also provides valuable quantitative binding information. Each of these methods has its limitations, such as being time-consuming or requiring considerable time and material. In addition, fluorescence polarization (FP),^11^ Förster resonance energy transfer (FRET) and time-resolved FRET (TR-FRET) detect molecular proximity and are compatible with plate-based HTS formats.^12^ However, these fluorescence-based methods require fluorophore labeling and assay optimization, and all are often time- and material-intensive, limiting their suitability for large-scale HTS.

Our laboratory has developed a multiwell, plate-based DNA-linked Inhibitor ANtibody Assay (DIANA), which offers a simple, rapid, and highly sensitive technique for protein detection, quantification, and HTS of protein-compound interactions. DIANA enables the determination of interaction strength from a single-well measurement^14^ and is suitable for both small- and large-scale HTS, enabling the screening of thousands of compounds per day. We have demonstrated that DIANA is an efficient method for screening compound libraries to identify new inhibitors for clinically relevant enzymes, including prostate-specific membrane antigen (PSMA),^14^ influenza neuraminidase (NA),^15^ influenza polymerase PB2,^16^ carbonic anhydrase IX,^17^ and fibroblast activation protein,^18^ as well as CD73.^19^ In this study, we present the development of DIANA for HTS of inhibitors targeting human PD-L1. This assay also enables the determination and comparison of dissociation constant (*K*_d_) for anti-PD-L1 agents. Our method allowed us to perform HTSs within a few hours on two compound libraries: an in-house IOCB library and the commercial OTAVA Chemicals peptide library.^20^ Additionally, we determined the *K*_d_ for the PD-L1 protein directly from HTS data and validated the method’s robustness by analyzing three FDA-approved anti-PD-L1 mAbs, as well as the anti-PD-L1 small molecule WL12 ^21^ and its natural receptor PD-1.

To the best of our knowledge, this work represents the first automated high-throughput screening study for human PD-L1. This study presents the first DIANA assay developed to block protein–protein interactions, in which a macrocyclic peptide was used for probe development.

## Results

### Design of DIANA Detection Probe

A target-specific detection probe is critical for the success of DIANA. As the component targeting PD-L1, we selected the macrocyclic peptide WL12.^21^ This compound combines the general advantages of macrocyclic peptides, such as more stability over their linear counterparts with the presence of a single primary amine, enabling efficient chemical conjugation to a DNA oligonucleotide.^22^ We utilized the modification of the primary amine to conjugate WL12 ^23^ to a DNA oligonucleotide via copper-catalyzed azide–alkyne cycloaddition, resulting in the formation of a PD-L1-targeted DIANA detection probe (see Methods section for further details).

### Optimization and Validation of DIANA

As a sandwich-based assay, DIANA begins with NeutrAvidin immobilized at the bottom of the wells. Recombinant human PD-L1 is then captured with biotinylated antihistidine-tagged iBodies developed by our team (Figure 1).^24^ Addition of the probe leads to target-specific binding and subsequent detection and quantification by qPCR (Figure 1). This method allows for coincubation of the probe with a test compound, enabling evaluation of inhibitory potency based on competitive binding. Comparing cycle number differences (ΔCq) between wells with and without the test compounds enables assessment of inhibition potency and determination of the inhibition constant in a single-well experiment (see Methods section for further details). Additionally, in the absence of test compounds, the amount of PD-L1 can be quantified via qPCR using a calibration curve.

**Figure 1:**
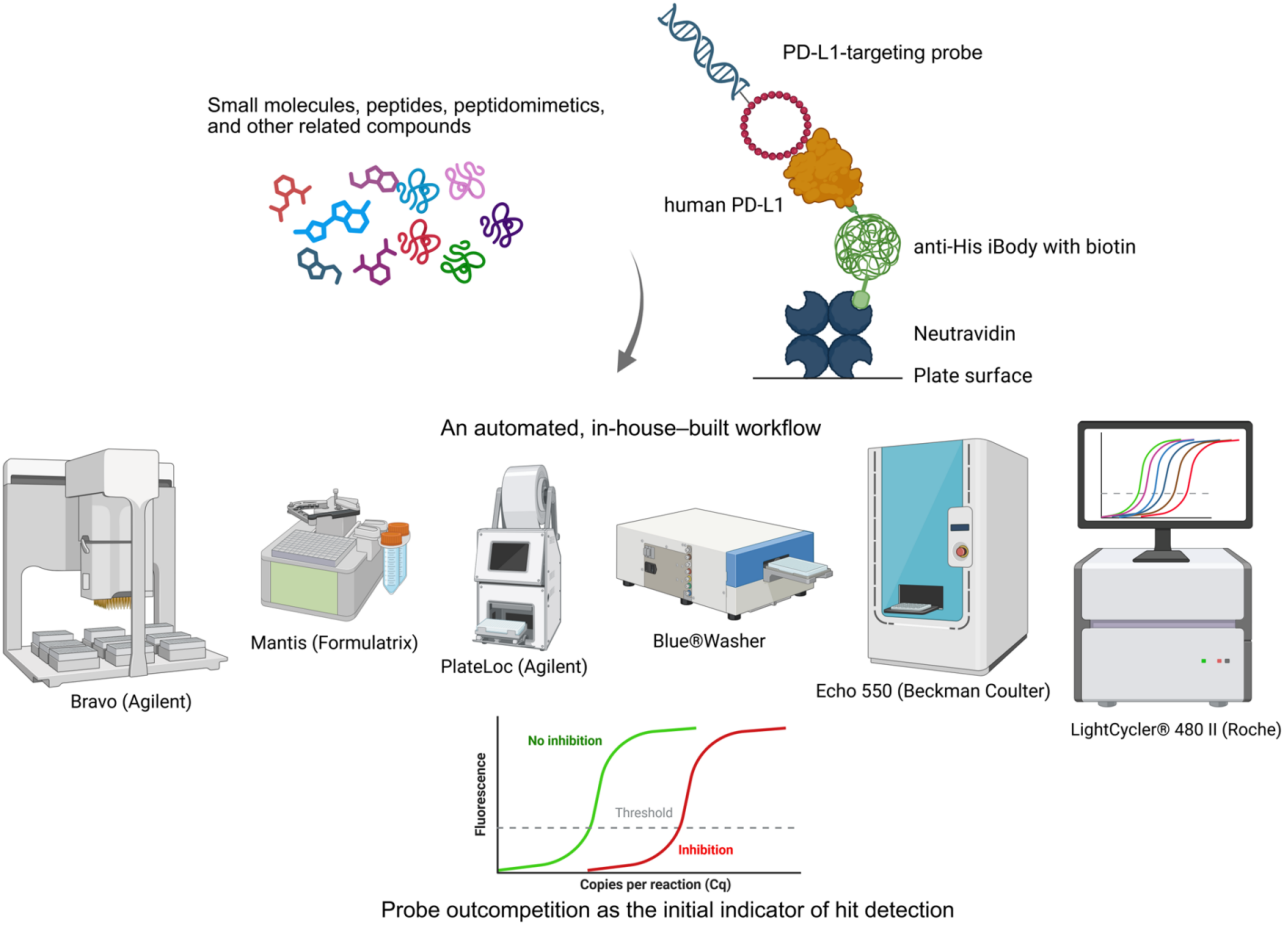
Schematic representation of HTS DIANA for evaluation of PD-L1-targeting compounds and the instruments used (created with BioRender.com). All pipetting steps were automated. Detailed information about the assay steps and instruments is available in the Methods section and in Table 4.

To optimize DIANA for PD-L1 ligand screening, we systematically evaluated various experimental conditions. We determined that the optimal working amounts per well are 185 pg PD-L1 and 200 pM probe. Additionally, because compound libraries are often dissolved in DMSO, we assessed the assay’s tolerance to 10% DMSO and found no significant impact on its performance. These optimizations yielded a broad assay window of approximately 13 qPCR cycles (spanning over 4 orders of magnitude).

To validate the DIANA setup, we measured the *K*_d_ values of various PD-L1-targeting compounds, including FDA-approved therapeutic antibodies, WL12 as a small molecule, and PD-1. Using serial dilutions of each compound, *K*_d_ values were calculated along with their standard deviations, as detailed in the Methods section. The therapeutic mAbs showed strong binding affinities with the lowest *K*_d_ values: atezolizumab (0.0023 ± 0.0001 nM), avelumab (0.007 ± 0.003 nM), and durvalumab (0.0024 ± 0.0003 nM). The macrocyclic peptide WL12 had a *K*_d_ of 0.26 ± 0.09 nM, while PD-1 exhibited a *K*_d_ of 3.7 ± 0.6 nM. These values generally align with previously reported trends in literature, as summarized in Table 1.

**Table 1:**
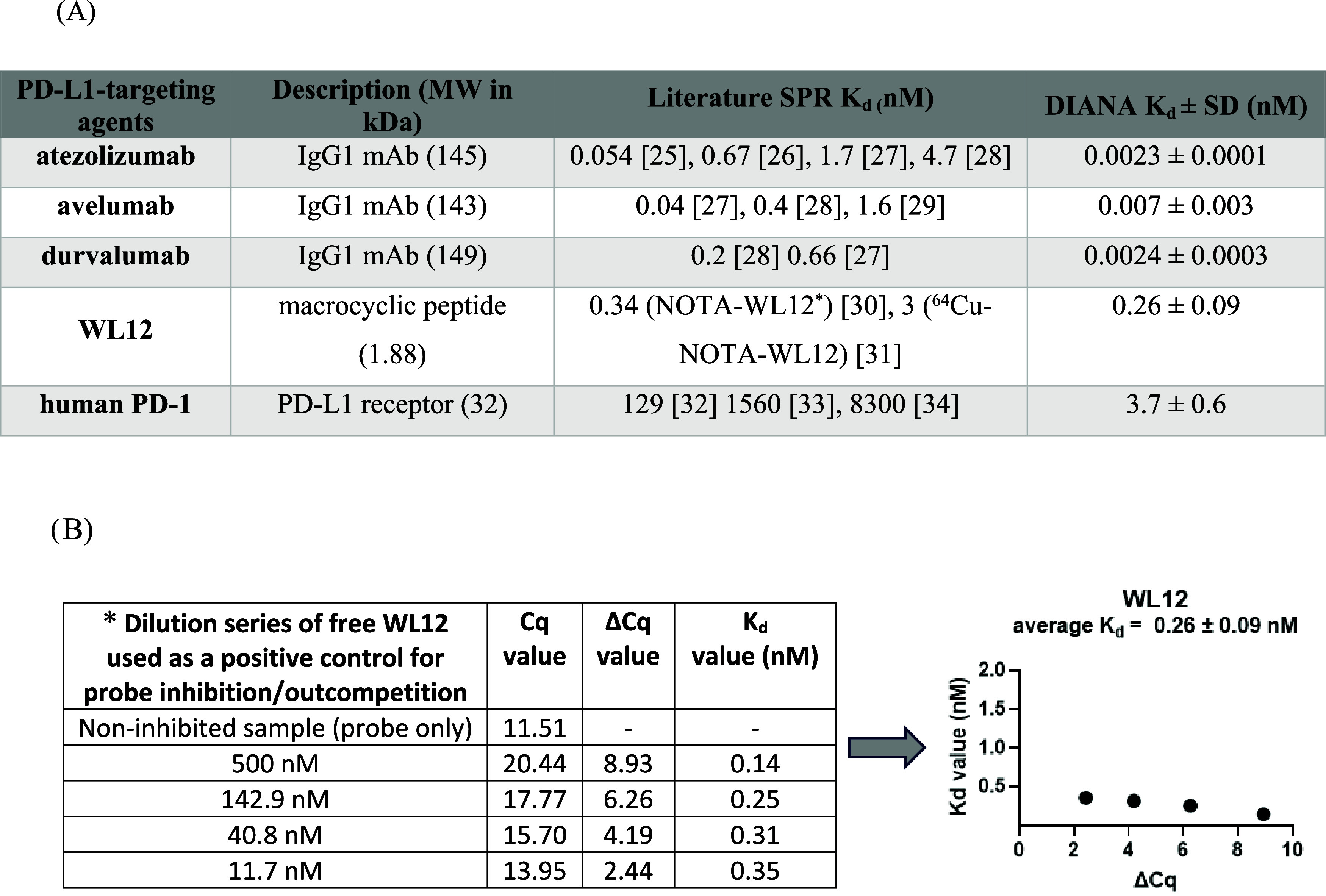
Validation of the Method by Comparison of *K*_d_ Values of PD-L1–Binding Agents Determined by DIANA with Previously Published Values Obtained Using SPR (A). Determination of the *K*_d_ Value for WL12, Used as a Positive Control (B)^25–34^

* WL12 is shown as an example to illustrate *K*_d_ determination for control molecules in competitive DIANA. Multiple dilutions of the control molecules were used to calculate the *K*_d_ value at each concentration point, resulting in an average *K*_d_ of 0.26 ± 0.09 nM. The procedures and calculations are detailed in the Methods section and in Navrátil et al.^14^

### HTS of the IOCB Compound Library

The IOCB library is an ever-expanding collection of small molecules developed at the Institute of Organic Chemistry and Biochemistry of the Czech Academy of Sciences. We tested 5,280 diverse and unique compounds, including peptides, peptidomimetics, nucleosides, nucleotide analogs, steroids, and natural compounds. The development of DIANA to screen the IOCB library for inhibition of various protein targets initially utilized a manual workflow in a 96-well plate format,^17^ which was well-suited for assay optimization and early stage experiments. However, as the method advanced, it was transitioned to an automated workflow using a 384-well pooled plate format. In this setup, each well contains a mixture of 11 individual compounds, each at a concentration of 900 μM (the final concentration of an individual compound in DIANA was 9 μM). This approach minimizes the number of plates needed and shortens the assay duration, while maintaining the integrity and reliability of the assay results with no significant interference.^17^

DIANA for PD-L1 ligand screening boasts a substantial assay window, defined by the difference in Cq values between noninhibited wells and the most inhibited wells, of 13 cycles—equivalent to more than 4 orders of magnitude. This broad assay window makes DIANA particularly advantageous for HTS, enabling evaluation of thousands of compounds without requiring serial dilution. The method’s robustness and reproducibility were confirmed by low variability in the control wells. Specifically, the standard deviation of the negative control, calculated from 24 identical noninhibited wells, was only 0.09 Cq and a *Z*′-factor of 0.94 (calculated from control wells during the pooled screen) qualifies the assay as excellent for HTS.^35^ During screening, WL12 was used as a positive control to inhibit the interaction between the probe and PD-L1. WL12 yielded a *K*_d_ of 0.26 ± 0.09 nM, based on 24 wells tested across six different concentration points.

To identify hits from the HTS of the pooled IOCB library, the hit selection threshold was stringently set at ΔCq ≥ 0.80, corresponding to more than eight times the standard deviation of the noninhibited wells. Any well exceeding this ΔCq threshold was classified as a potential hit.

To detect assay interference in the IOCB library, we employed a counter-screen in which a high concentration of WL12 saturates PD-L1 binding. Under these conditions, true inhibitors should show no additional ΔCq change in the counter-screen for any tested compound, whereas assay interferers (e.g., chelators or PCR inhibitors) would continue to alter ΔCq even in the presence of WL12. The counter-screen was therefore used to identify and exclude false positives arising from assay interference. All pooled wells were retested in the presence of 83 nM WL12. Wells exhibiting a significant change in Cq values during the counter-screen were classified as assay interferers and removed from the hit list.

As each pooled well contains 11 individual compounds, a deconvolution process was necessary to determine the specific compound(s) responsible for each verified hit. The deconvolution procedure was similar to the initial screening but involved testing each compound individually at a concentration of 10 μM.

The assay window for the deconvolution experiment remained comparable to that observed during screening, measuring 12.54 cycles (spanning more than 4 orders of magnitude), with a standard deviation of 0.19 cycles from 16 wells containing no compounds. The positive control WL12 exhibited a *K*_d_ of 0.13 ± 0.02 nM in the deconvolution experiment, consistent with the *K*_d_ obtained during the screening experiment. All hits from the pooled library were successfully confirmed after deconvolution, with two individual compounds identified from a single well.

Using this approach, we initially identified 24 weak hits from the pooled library screening. Four of these compounds passed the counter screen step (Figures 2 and 3). Further testing in a relevant PD-1/PD-L1 cellular assay revealed that these compounds were false positives (Figure 2).^23^ Upon closer investigation, we determined that the false positives were compounds resembling a chelator moiety, which likely interfered with the ion^36^ involved in PD-L1 immobilization during DIANA, where the TrisNTA is used to bind to the his-tag of PD-L1 protein (Figure 1). As a result, these compounds were excluded from further processing.

**Figure 2:**
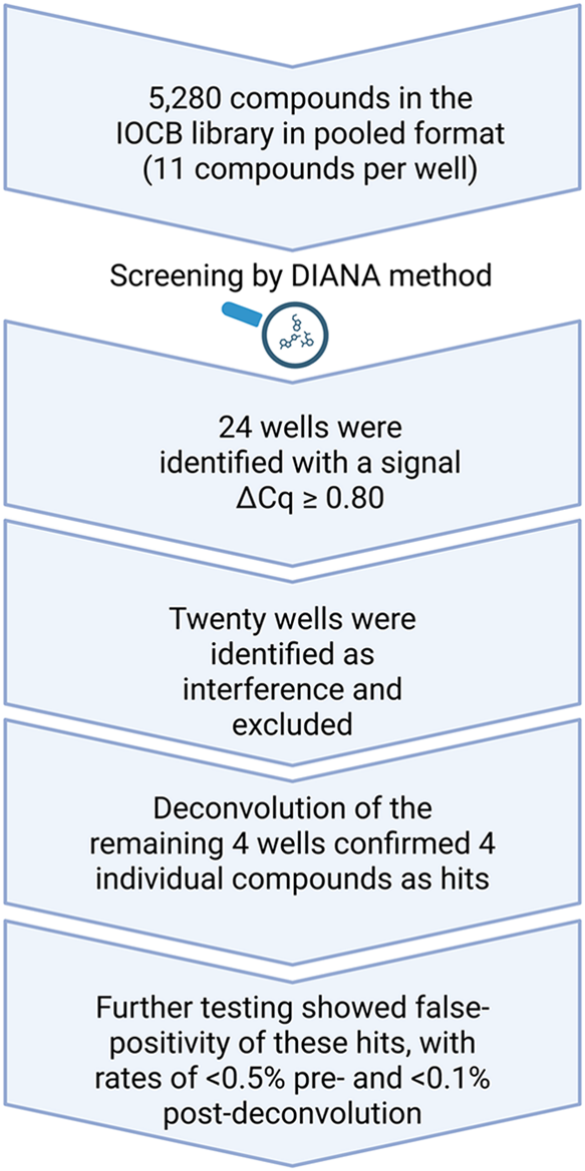
Schematic representation of HTS process of the IOCB library in pooled format by DIANA method. Created in BioRender. Cermakova, K. (2025) https://BioRender.com/ozmkslp.

**Figure 3:**
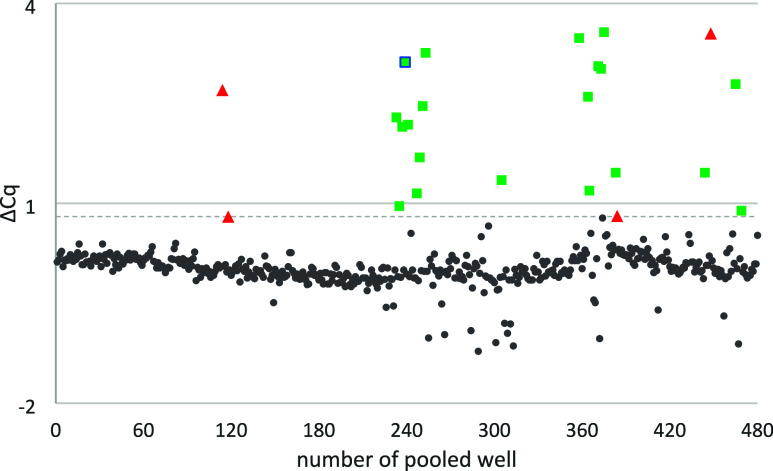
Screening of the pooled IOCB library against PD-L1 with DIANA. Each pooled well is represented as a dot along the *x*-axis, corresponding to its position number. The ΔCq on the *y*-axis indicates the cycle difference between the wells tested with compounds and the average of wells without any compounds. The dashed line represents the established cutoff threshold for hit identification. Results from screening of the pooled library are shown, with values under the cutoff represented in gray, including interferences (in green). Four identified hits (in red) were confirmed by DIANA after excluding other interferences. WL12 was tested as a positive control at six concentration points in duplicate on each plate (24 total data points). These measurements yielded an average *K*_d_ of 0.26 ± 0.09 nM, with the maximum tested WL12 concentration corresponding to a ΔCq of 13.03. Cq values from noninhibited wells pooled across all plates (24 points) showed an average Cq of 9.89 ± 0.14, which was defined as ΔCq = 0.

The sequence of major events in the HTS process of the IOCB library in pooled format using the DIANA method is summarized in Figure 2.

### HTS of a Commercial Peptide Library

The α-Helix peptidomimetic library (Otava Chemicals) is a commercially available collection containing 1,298 natural and non-natural small molecules that mimic α-helical structures. It was primarily developed for drug discovery research, focusing on targeting protein–protein interactions that are critical for biological functions.^20^ While the library was initially packaged in 96-vial boxes, we reformatted it into a 384-well plate format to better suit our HTS method. Screening of the α-helix peptidomimetic library followed a similar protocol to that used for the IOCB compound library. Due to the lower number of compounds in the peptidomimetic library, pooling multiple compounds in single wells was not required. Instead, the screening was conducted using a compound-by-compound testing approach similar to the deconvolution experiment with the IOCB library.

In the HTS DIANA of the α-helix peptidomimetic library, the assay window reached 12.63 cycles (spanning more than 4 orders of magnitude), comparable to that observed in the screening of the IOCB library. The standard deviation of the 24 identical noninhibited wells used to calculate ΔCq was 0.18 Cq. WL12 served as a positive control, yielding a *K*_d_ of 0.32 ± 0.05 nM (average of 24 wells across six different concentration points). To identify potential hits, the cutoff threshold was strictly set at three times the standard deviation of the noninhibited wells, or 0.66 ΔCq. Screening of the commercial α-helical peptidomimetic library yielded a *Z*′-factor of 0.94, demonstrating the suitability of DIANA as a reliable high-throughput screening (HTS) method.^35^ Compounds exhibiting a ΔCq greater than 0.66 were considered as potential hits. HTS of the α-helix peptidomimetic library yielded six very weak initial hits (Figure 4 and Table 2). However, upon retesting the compounds in a separate DIANA assay, no inhibition was observed, which may be due to the low technical variation in Cq values between the automated HTS and the manual, smaller-scale experiment.

**Figure 4:**
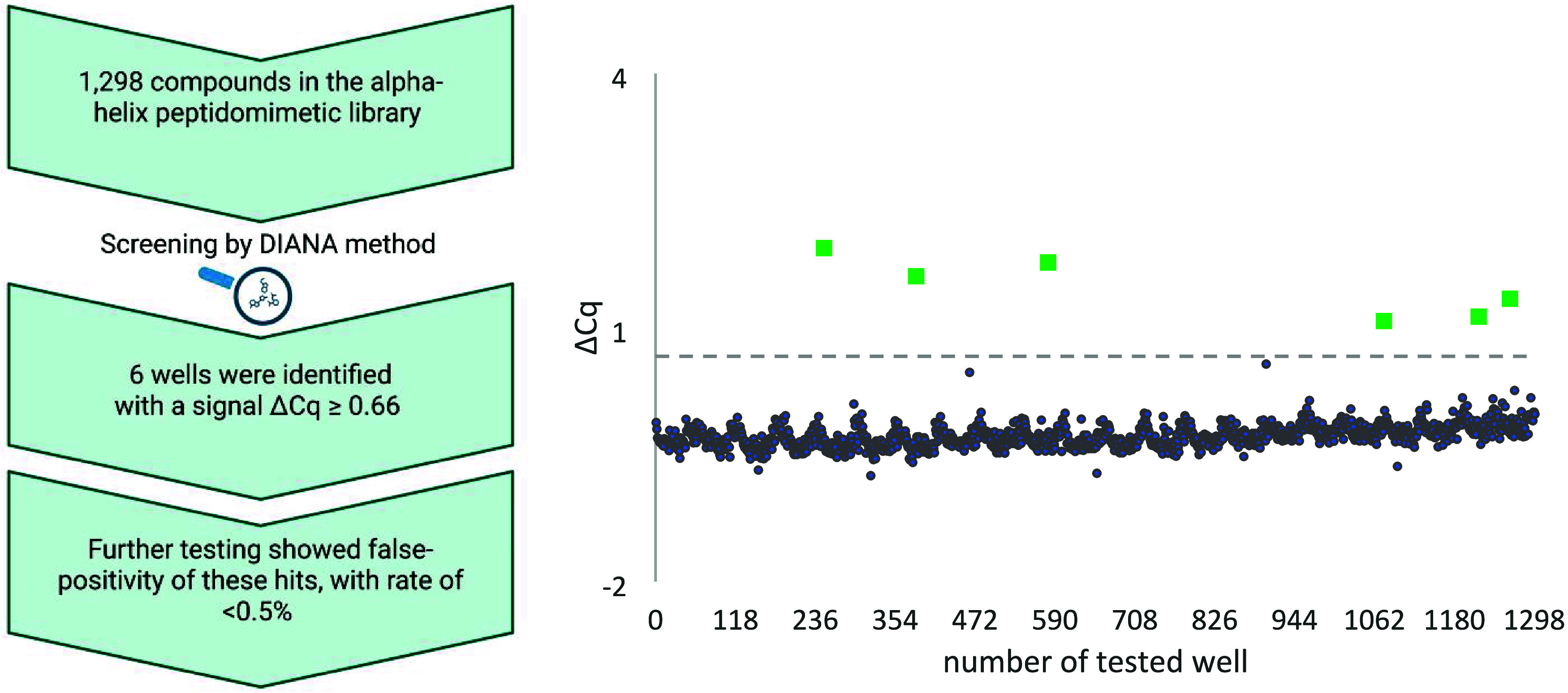
Screening of the commercial α-helical peptidomimetic library against PD-L1 using the DIANA assay. Each compound tested is represented as a dot on the *x*-axis. The ΔCq on the *y*-axis indicates the cycle difference between the wells tested with compounds and the average of the wells without any compounds. The dashed line represents the established cutoff threshold for hit identification. Results from the screening of the library are shown as six identified hits (in green), and other compounds with values under the cutoff represented in gray. Each tested plate included the positive control WL12 at six concentration points in duplicate (24 total wells), yielding an average *K*_d_ of 0.32 ± 0.05 nM; the maximum tested WL12 concentration corresponded to a ΔCq of 12.63. Negative control wells (noninhibited; 12 per plate, 24 total wells) produced an average Cq of 10.22 ± 0.18.

**Table 2:**
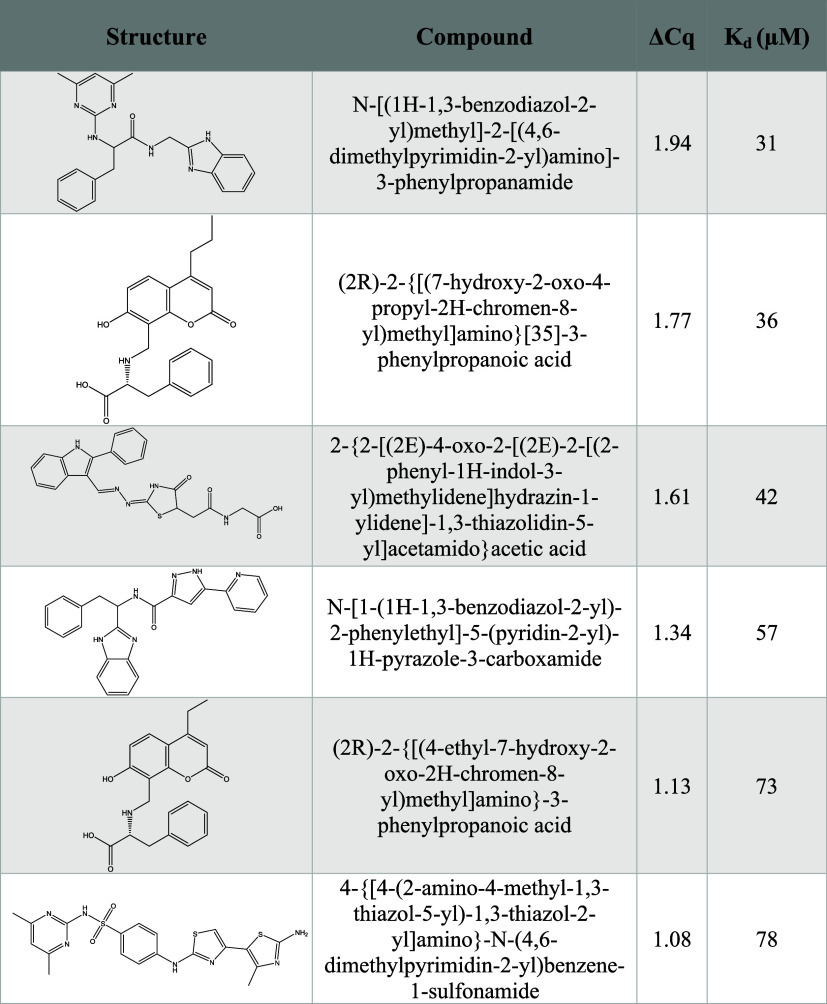
Hit List of Compounds Identified from the Commercial α-Helix Peptidomimetic Library Screening

Comparison of the screening parameters of the IOCB and α-helical peptidomimetic libraries by DIANA in HTS format is shown in Table 3.

**Table 3:** Comparison of the Screening Parameters of the IOCB and α-Helical Peptidomimetic Libraries by DIANA in HTS Format^a^

	pooled IOCB library screening (from deconvolution)	peptidomimetic library
Screened compounds	5,280	1,298
Average Cq of noninhibited control wells	9.89 ± 0.14 (10.31 ± 0.19)	10.22 ± 0.18
Average Cq of the most inhibited control wells	22.92 ± 0.11 (22.86 ± 0.04)	22.85 ± 0.18
Assay window (Cq)	13.03 (12.55)	12.63
*K*_d_ values for the positive control, WL12 (nM)	0.26 ± 0.09 (0.13 ± 0.02)	0.32 ± 0.05
Interplate *Z*′-factor	0.94	0.94

a The parameters include: the number of compounds in the tested library; assay window (the difference in Cq values between non-inhibited wells and the most inhibited wells); positive control, defined as the average *K*_d_ value of WL12 across all tested plates; and *Z*′-factor, calculated as the interplate control across all tested plates.

## Summary of the Results

DIANA-based high-throughput screening assay for human PD-L1 exhibits a broad dynamic range (>4 orders of magnitude), robustness (*Z*′-factor = 0.94), and high DMSO tolerance (10%). Validation with FDA-approved antibodies, the macrocyclic peptide WL12, and PD-1 yielded dissociation constants consistent with reported binding trends. Screening of two compound libraries identified only weak, nonconfirmed hits, underscoring both the stringency of the platform and the suitability of DIANA as a robust quantitative tool for PD-L1 ligand discovery.

## Methods

### Cloning, Expression, and Purification of Recombinant Human PD-L1

DNA encoding the extracellular domain (residues 19–239) of human PD-L1 was amplified by PCR from the pCMV3 plasmid containing full-length human PD-L1. The detailed procedure has been previously described.^23^

### Preparation of WL12 Modified with PEG-4-N_3_

N_3_–PEG-3-OCH_2_CH_2_COOSu (2.5 mg, 6.4 μmol, 1.2 equiv) and DIEA (20 μL, excess) were dissolved in MeCN:H_2_O/10:1 (1 mL). Then, WL12 (10 mg, 5.3 μmol, 1.0 equiv) was added. The resulting mixture was stirred for 1 h at room temperature. The product was purified by RP C18 HPLC (gradient 30–80% MeCN in H_2_O + 0.1% TFA) to obtain WL12 modified with PEG-4-N_3_ linker as a TFA salt (4.5 mg, 38%).^23^

### Preparation of DIANA Detection Probe

The oligonucleotide 5′-GAA ACT GGA CCC CAG GGT GGT TTC CTG CCA GTT GAG CAT TTT TAT CTG CCA CCT TCT CCA CCA GAC AAA AGC TGG T-C8-alkyne-dT-T-3′ (Generi Biotech) was conjugated with the PD-L1-binding macrocyclic peptide WL12, which contains an azide (−N_3_) group, using copper-catalyzed azide–alkyne cycloaddition. The conjugation reaction was carried out with a 5-fold molar excess of azide-modified WL12 (250 μM) over alkyne-modified oligonucleotide (50 μM). The reaction mixture included 5 mM aminoguanidine hydrochloride, 8 mM BTTP, 5.9 mM CuSO_4_, and 20 mM sodium ascorbate in HEPES buffer, pH 7.0. The mixture was incubated at 37 °C for 3 h. The final conjugate (DIANA probe) was then purified from the unconjugated small molecule by ultrafiltration using a 10 kDa cutoff (UltraCell 0.5 mL 10K, Merck) and analyzed by liquid chromatography–mass spectrometry.

### DIANA Protocol for PD-L1 Inhibition Screening

The general DIANA assay protocol has been described by Navrátil et al.^14^ In this study, we optimized the setup with some modifications (Table 4). First, NeutrAvidin (NeutrAvidin Protein, Cat. No. 31050, Thermo Fisher Scientific) was directly immobilized onto the bottom of the wells (FrameStar 96-Well Semi-Skirted PCR Plate, Cat. No. 4ti-0951, for 96-well plates, or FrameStar 384-Well Skirted PCR Plate, Cat. No. 4ti-0381, for 384-well plates; both from 4titude Ltd., Berlin, Germany) by adding 5 μL of a 10 ng/mL solution in TBS (Tris-buffered saline: 20 mM Tris–HCl, 150 mM NaCl, pH 7.5). The wells were blocked with 100 μL of 1.1% casein blocker (SDT-reagents, Cat. No. CBC1, Stereospecific Detection Technologies GmbH) in TBS overnight, followed by washing by TBST (20 mM Tris–HCl, 150 mM NaCl, 0.05% (w/v) Tween 20). Next, 100 nM anti-His iBody in TBST′ (20 mM Tris–HCl, 150 mM NaCl, 0.1% (w/v) Tween 20) was applied to the bottom of each well and incubated for 1 h, followed by washing with TBST′. Afterward, 5 μL of 37 pg/μL His-tagged PD-L1 protein in TBST′ was added to each well for 1 h, followed by washing. Then, 5 μL of 200 pM detection probe, diluted in TBST′ with 0.011% (v/v) casein, was mixed in a 9:1 ratio with an inhibitor dissolved in 100% DMSO (Sigma-Aldrich, Cat. No. 472301–100 ML) and added to the bottom of each well (final concentration of 10% DMSO) for 1 h. The plate was washed, and the amount of bound probe was determined by qPCR as described previously.^14^

In HTS format (Table 4), the same amounts of reagents were used in 384-well plates. A Bravo Automated Liquid Handling Platform (Agilent Technologies, Santa Clara, CA, USA) added NeutrAvidin, anti-His iBody, PD-L1 protein, and the probe, while casein blocker and master mix were applied by Mantis (Formulatrix Inc., Bedford, MA, USA). The inhibitors were transferred in nanoliters from libraries using a Labcyte Echo 550 liquid handler (Beckman Coulter, Inc., Brea, CA, USA). The rest of the DMSO was filled with probe buffer up to 10% DMSO in the final solution. Washing steps were carried out using the automated BlueWasher (BlueCatBio, Concord, MA, USA). A PlateLoc Thermal Microplate Sealer (Agilent Technologies, Inc., Santa Clara, CA, USA) was used to seal the plates with a cover during incubations. All procedures followed the DIANA protocol described above (Table 4). During screening, plates were processed sequentially. Screenings were performed in singlicate; however, hit confirmation and *K*_d_ determination were conducted in duplicate. All incubation steps, including blocking step, were performed at room temperature (22–25 °C).

**Table 4:** DIANA Method Protocol Used for Ligand Testing in 96-Well Plates and for High-Throughput Screening in 384-Well Plates against PD-L1^a^

step	parameter	volume (μL)/ 96-well plates	volume (μL)/ 384-well plates	time	amount or concentration/well	description	instrument
1	NeutrAvidin immobilization	5	5	1 h	50 ng	Nonspecific absorption on assay plate	Bravo (Agilent)
2	Blocking (1:5 diluted casein)	100	20	>16 h	---	Blocking of nonspecific binding sites	Mantis (Formulatrix)
3	Wash	150	30	3 × 1 min	---	Removal of unbound material	BlueWasher
4	Anti-His iBody	5	5	1 h	100 nM	Capture reagent for His-tagged rPD-L1	Bravo (Agilent)
5	Wash	150	30	3 × 1 min	---	Removal of excess capture reagent	BlueWasher
6	His-tagged rhPD-L1	5	5	1 h	185 pg	Target protein immobilization	Bravo (Agilent)
7	Wash	150	30	3 × 1 min	---	Removal of excess rhPD-L1	BlueWasher
8	Detection probe ± inhibitor	5	5	1 h	200 pM	Competitive binding step	Probe: Bravo (Agilent); Compounds: Echo 550 (Beckman Coulter)
9	Wash	150	30	4 × 1 min	---	Removal of excess probe	BlueWasher
10	PCR master mix	5	5	added immediately before qPCR	According to manufacturer’s protocol	qPCR reaction setup	Mantis (Formulatrix)
11	Assay read-out	---	---	---	---	Quantification of bound probe	LightCycler 480 II (Roche)

a The schematic representation of the instruments is shown in Figure 1, and more detailed information about the instrument manufacturers is provided in the Methods section. All incubation steps were performed at room temperature (22–25 °C).

For qPCR, LightCycler 480 SYBR Green I Master (Cat. No. 04887352001, Roche Diagnostics, Basel, Switzerland) was used according to the manufacturer’s instructions and supplemented with forward and reverse primers at a final concentration of 1 μM each (5′-CCA GCT TTT GTC TGG TGG AG-3′ and 5′-CCT GCC AGT TGA GCA TTT TT-3′). qPCR was performed with an initial denaturation at 95 °C for 3 min, followed by 45 cycles of 10 s at 95 °C, 30 s at 66 °C, and 30 s at 72 °C, and a final incubation at 37 °C for 2 min, using a LightCycler 480 II qPCR Instrument (Roche Diagnostics, Basel, Switzerland). Excitation and emission filters were set to 465 and 510 nm, respectively. Threshold cycle (Cq) values were determined from fluorescence amplification curves using the maximum second-derivative method implemented in the LightCycler 480 II Software (Roche).

### Determination of Inhibition Constants by DIANA

One key feature of DIANA is the ability to determine a compound’s *K*_d_ value in the presence of a detection probe from a single well measurement. In this study, *K*_d_ was determined according to the following equation: Compound *K*_d_ = (2^–ΔCq^/(1 – 2^–ΔCq^)) × *I*_tot_/(1 + (*P*_tot_/Probe *K*_d_)),^14^ where the ΔCq is the difference between Cq values of the well(s) in the presence of the compound and the mean Cq of wells without the compound. *I*_tot_ represents the total compound concentration, *P*_tot_ represents the total probe concentration (200 pM), and *K*_d_ is the dissociation constant of the probe determined by incubating serial dilutions of the probe with a constant amount of PD-L1 (*K*_d_ = 1.32 nM).

## Discussion

Given the complexity of the PD-1/PD-L1 interaction and the limitations of existing antibody-based therapies, advancements in assay technologies are essential for the discovery of next-generation immunotherapeutics.^37^

One of the major challenges in this field is the identification of small-molecule inhibitors that can effectively disrupt the large, flat, and hydrophobic interface of PD-1/PD-L1—an interface that spans approximately 1970 Å^2^ and lacks well-defined binding pockets.^38,39^

Surface plasmon resonance (SPR) has been used to study binding interactions, but its application in high-throughput screening (HTS) is limited by throughput and the need for protein immobilization. Techniques such as isothermal titration calorimetry (ITC) and microscale thermophoresis (MST) provide quantitative binding information but are material- and time-intensive. In contrast, techniques like Fluorescence Polarization (FP), which measures changes in molecular rotation, and Förster Resonance Energy Transfer (FRET) and its time-resolved variant (TR-FRET), which detect proximity through energy transfer, are inherently compatible with automated high-throughput screening.^40^ However, this compatibility comes at the cost of requiring the covalent attachment of fluorophores and extensive assay development. These limitations highlight the need for methods that combine quantitative binding with higher-throughput compatibility. In contrast, the DNA-linked Inhibitor Antibody Assay (DIANA) uses small-molecule ligands covalently linked to DNA, enabling detection by qPCR with extremely high sensitivity, minimal protein and ligand consumption, and the capacity to process vast libraries in parallel. DIANA does not require protein labeling, thus it reduces assay artifacts and can detect very low-abundance binding events, making it particularly advantageous for PD-L1 screening when throughput, sensitivity, and material efficiency are critical.^41–43^

In this study, we present DIANA as a sensitive, robust, and scalable HTS platform tailored to circumvent some of these limitations. DIANA enables rapid testing of tens of thousands of compounds per day with minimal sample consumption. Its major innovation lies in the ability to determine dissociation constants (*K*_d_) directly from single-well measurements via competitive binding—eliminating the need for serial dilutions and significantly accelerating quantitative profiling of binding affinities.^36^

We demonstrated that DIANA offers a broad dynamic range spanning more than 4 orders of magnitude and achieves excellent assay quality, with the interplate *Z*′-factor above 0.9. The assay was validated using known PD-L1 binders, including three FDA-approved mAbs (atezolizumab, avelumab, and durvalumab), the macrocyclic peptide WL12, and the native PD-1 receptor. Moreover, the assay demonstrated excellent tolerance to DMSO (up to 10%), a key feature for compatibility with most small-molecule libraries. Interestingly, we observed very consistent and reproducible Cq values in the key control elements of the method—specifically in the most inhibited and uninhibited control wells—during the screening and counter-screening of the IOCB library, as well as in the screening of the peptidomimetic library, all of which were performed in independent experiments (Table 3). We also observed a general correlation between the *K*_d_ values obtained by DIANA and previously reported data, although some deviations in absolute values were present, supporting the potential of the method. The differences in absolute *K*_d_ values, despite consistent binding trends, can be attributed to variations in assay methods (e.g., DIANA vs other methods such as SPR), buffer conditions, temperature, and detection sensitivity. The observed differences likely reflect methodological distinctions between DIANA and techniques such as SPR. DIANA measures competitive equilibrium binding in solution with qPCR-based detection, whereas SPR relies on kinetic measurements using surface-immobilized proteins. The high sensitivity of the qPCR readout enables detection of low fractional occupancies that may be more difficult to capture by SPR. In SPR experiments, factors including immobilization chemistry, surface density, protein orientation, protein quality, glycosylation, and ligand integrity can influence apparent affinity. In addition, DIANA’s detection mechanism and binding model assumptions may also affect the measured values. As shown in Table 1, reported SPR *K*_d_ values for the same antibody–PD-L1 interactions span more than 2 orders of magnitude, reflecting differences in experimental setups and methodologies across the literature. Accordingly, some variation is expected, and despite differences in absolute *K*_d_ values, the consistent relative affinity ranking between DIANA and SPR supports the robustness and validity of the assay.

The high-throughput capacity of DIANA was further exemplified by the screening of over 5,200 compounds from the IOCB in-house library and 1,298 compounds from the commercial α helix peptidomimetic library. In the IOCB screen, the use of pooled formats (11 compounds per well) allowed for significant resource savings, while the single-compound format used for the peptidomimetic library demonstrated DIANA’s versatility across different screening workflows. Although initial screenings yielded 24 potential hits from the IOCB library, only four were not excluded in counter-screening, and none were ultimately validated in cellular PD-1/PD-L1 assays. Similarly, six weak hits from the peptidomimetic screen were determined to be false positives during hit confirmation.

Despite the absence of confirmed biological hits, these results reflect more on the limitations of the screened libraries—particularly their size and chemical diversity—than the performance of DIANA itself. Indeed, the low false-positive rates we observed (<0.5% predeconvolution and <0.1% postdeconvolution) underscore the specificity and precision of the platform. DIANA’s design also enables effective identification of assay interference through the integration of a counter-screening step with the known PD-L1 ligand WL12, providing a built-in safeguard against false positives.

In conclusion, DIANA stands out as a powerful, high-throughput platform ideally suited for drug discovery targeting challenging protein–protein interactions such as PD-1/PD-L1. It provides distinct advantages over conventional methods, including rapid throughput, single-well *K*_d_ determination, minimal sample requirements, and compatibility with both pooled and individual compound formats.

Moving forward, we plan to expand DIANA-based screening to include a broader and more chemically diverse set of libraries—both commercial and academic—to enhance hit discovery rates. Additionally, integration with fragment-based drug discovery and structure-guided optimization may further increase the likelihood of identifying viable small-molecule inhibitors.^44^
